# Classical Harmony and Separability

**DOI:** 10.1007/s10670-018-0032-6

**Published:** 2018-07-30

**Authors:** Julien Murzi

**Affiliations:** University of Salzburg, Salzburg, Austria

## Abstract

According to logical inferentialists, the meanings of logical expressions are fully determined by the rules for their correct use. Two key proof-theoretic requirements on admissible logical rules, harmony and separability, directly stem from this thesis—requirements, however, that standard single-conclusion and assertion-based formalizations of classical logic provably fail to satisfy (Dummett in The logical basis of metaphysics, Harvard University Press, Harvard, MA, 1991; Prawitz in Theoria, 43:1–40, 1977; Tennant in The taming of the true, Oxford University Press, Oxford, 1997; Humberstone and Makinson in Mind 120(480):1035–1051, 2011). On the plausible assumption that our logical practice is both single-conclusion and assertion-based, it seemingly follows that classical logic, unlike intuitionistic logic, can’t be accounted for in inferentialist terms. In this paper, I challenge orthodoxy and introduce an assertion-based and single-conclusion formalization of classical propositional logic that is both harmonious and separable. In the framework I propose, classicality emerges as a structural feature of the logic.

According to logical inferentialists, the meanings of logical expressions are fully determined by the rules for their correct use. Two key proof-theoretic requirements on admissible logical rules, harmony and separability, directly stem from this thesis—requirements, however, that standard single-conclusion and assertion-based formalizations of classical logic provably fail to satisfy (Dummett 1991; Prawitz 1977; Tennant 1997; Humberstone and Makinson 2011). On the plausible assumption that our logical practice is both single-conclusion and assertion-based, it seemingly follows that classical logic, unlike intuitionistic logic, can’t be accounted for in inferentialist terms. In this paper, I challenge orthodoxy and introduce an assertion-based and single-conclusion formalization of classical propositional logic which is both harmonious and separable. In the framework I propose, classicality emerges as a structural feature of the logic.

Section 1 provides some background. Section 2 introduces the inferentialist argument against classical logic. Sections 3–6 present a novel axiomatisation of classical logic and prove that it is both harmonious and separable.^1^ Section 7 responds to some possible objections. Section 8 concludes.

## Harmony and Separability

Logical inferentialists typically contend that some basic inference rules are, in Michael Dummett’s terminology, self-justifying, in that they fully determine the meanings of the expressions they either eliminate or introduce. As Dummett puts it:

we are entitled simply to stipulate that [self-justifying laws] shall be regarded as holding, because by so doing we fix, wholly or partly, the meanings of the logical constants that they govern. (Dummett 1991, p. 246)

On their most common interpretation, introduction rules in a natural deduction system (henceforth, I-rules) state the sufficient, and perhaps necessary, conditions for introducing dominant operators in conclusions (in inferentialist parlance, the *canonical grounds* for introducing such conclusions); elimination rules (henceforth, E-rules) tell us what can be legitimately deduced from sentences containing dominant occurrences of logical operators. Logical inferentialism, then, becomes the claim that the meanings of logical expressions are fully determined by their I- and E-rules.^2^

As is well known, not any pair of I- and E-rules can determine the meaning of a logical expression, if ill-behaved connectives such as Prior’s tonk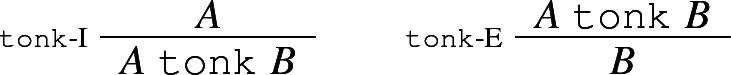 are to be ruled out (see Prior 1960). If the consequence relation is transitive, and at least one theorem can be proved, then *any* sentence can be proved. The inventor of natural deduction, Gerhard Gentzen, first sketched a solution to the problem. In a famous passage, Gentzen writes:

To every logical symbol&, $\vee , \forall , \exists , \rightarrow , \lnot$, belongs precisely one inference figure which ‘introduces’ the symbol—as the terminal symbol of a formula—and which ‘eliminates’ it. The fact that the inference figures&-E and $\vee$-I each have two forms constitutes a trivial, purely external deviation and is of no interest. The introductions represent, as it were, the ‘definitions’ of the symbols concerned, and the eliminations are no more, in the final analysis, than the consequences of these definitions. This fact may be expressed as follows: in eliminating a symbol, we may use the formula with whose terminal symbol we are dealing only ‘in the sense afforded it by the introduction of that symbol’. (Gentzen 1969, p. 80)

Gentzen argues that the I-rules of his newly invented calculus of natural deduction ‘fix’, or ‘define’, the meanings of the expressions they introduce. He also observes that, on this assumption, E-rules cannot be chosen randomly. They must be justified by the corresponding I-rules: they are, in some sense, their ‘consequences’. This key thought expresses *in nuce* the idea that I- and E-rules must be, in Dummett’s phrase, in *harmony* with each other. Conversely, if it is thought that E-rules are meaning-constitutive, I-rules cannot be chosen arbitrarily either (see e.g. Dummett 1991, p. 215).

This intuitive idea can be spelled out in a number of ways. Dummett (1991, p. 250) and Prawitz (1974, p. 76) define harmony as the possibility of eliminating *maximum formulae*, that is, formulae that occur both as the conclusion of an I-rule and as the major premise of the corresponding E-rule (see also Prawitz 1965, p. 34).^3^ The following reduction procedure for $\rightarrow$, for instance, shows that any proof of *B* via $\rightarrow$-I and $\rightarrow$-E can be converted into a proof from the same or fewer assumptions that avoids the unnecessary detour through (the introduction and elimination of) $A \rightarrow B$.

### Example 1

($$\rightarrow $$-*reduction*) 
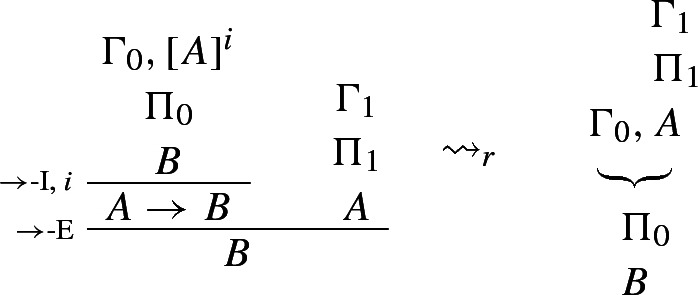


where $\leadsto _r$ reads ‘reduces to’.

Dummett (1991, p. 250) calls the availability of such procedures *intrinsic* harmony. He correctly points out, though, that intrinsic harmony only prevents E-rules from being stronger than the corresponding introductions, as in the case of Prior’s tonk. It does not rule out the possibility that they be, so to speak, too weak (see 1991, 287).^4^ A way to ensure that E-rules be strong enough is to require that they allow one to *reintroduce* complex sentences, as shown by the following *expansion*:

### Example 2

($$\rightarrow $$-*expansion*) 
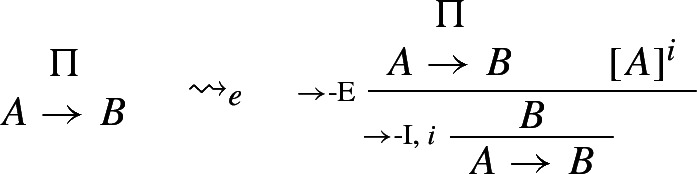
 where $\leadsto _e$ reads ‘expands to’.

This shows that any derivation $$\Pi $$ of $$A \rightarrow B$$ can be expanded into a longer derivation which makes full use of both $$\rightarrow $$-I and $$\rightarrow $$-E. The availability of an expansion procedure for a pair of I- and E-rules is sometimes referred to as *local completeness*. Accordingly, a pair of I- and E-rules for a constant $ can be taken to be harmonious * tout court* (or, in Dummett’s terminology, ‘stable’), if and only if there exist both reduction and expansion procedures for $-I and $-E. Alternative conceptions of harmony are developed in e.g. Read (2000) and Tennant (1997, 2008).^5^

But *why* should logical expressions be governed by harmonious rules? One motivating thought behind the requirement of harmony is that logic is *innocent*: it shouldn’t allow one to prove atomic sentences that we couldn’t otherwise prove (Steinberger 2009a). Yet another motivating thought has it that I-rules determine, in principle, necessary and sufficient conditions for introducing dominant occurrences of logical operators. For this reason, the thought goes, E-rules should ‘give us back’ the grounds specified by the corresponding I-rules, on the assumption that such grounds are in principle necessary (see e.g. Moriconi and Tesconi 2008, p. 105 and ff). This is in effect what Dummett calls the Fundamental Assumption, that ‘[i]f a statement whose principal operator is one of the logical constants in question can be established at all, it can be established by an argument ending with one of the stipulated I-rules’ (Dummett 1991, p. 251). The Assumption lies at the heart of the proof-theoretic accounts of validity (Prawitz 1985; Dummett 1991). As Prawitz puts it,

it is the whole [inferentialist] project that is in danger when the fundamental assumption cannot be upheld. (Prawitz 2006, p. 523)

If harmony is a necessary condition for logicality, Prior’s challenge is easily met: the tonk rules are spectacularly disharmonious, and hence cannot define a *logical* connective.^6^ But the tonk rules are also *non-conservative*: they allow one to prove sentences in the tonk-free language that were not previously provable in the absence of the rule for tonk (indeed they allow one to prove any such sentence). And indeed, the first response to Prior’s tonk, published by Nuel Belnap in 1962, was precisely that admissible rules should yield conservative extensions of the base systems to which they may be added.^7^

The demand for conservativeness is equivalent to the requirement that an admissible logical system be *separable*, i.e. such that every provable sentence or rule in the system has a proof that only involves either structural rules or rules for the logical operators that figure in that sentence or rule. This requirement is sometimes motivated by the further inferentialist thesis that to understand a linguistic expression is to know its role in inference (Boghossian 2003), i.e. to be able in principle to derive all correct uses of any logical expression one understands. Given separability, the totality of uses of $ (i.e. the derivations of rules and theorems involving sentences with $ as their main logical operator) is derivable from the basic rules for $, and, given the inferentialist account of understanding, one’s grasp of $’s rules is thereby sufficient for knowing $’s meaning.

Logical inferentialists typically assume an *atomistic* conception of our understanding of logical expressions. That is, they assume that in principle a speaker could understand e.g. $\wedge$ without understanding $\exists , \rightarrow$ without understanding $\lnot$, and so forth. Thus, Kent Bendall writes that ‘the order in which [...] logical rules are introduced should not matter’ (Bendall 1978, p. 255), since ‘it should not matter in what order one learns [...] the logical operators’ (Tennant 1997, p. 315). In a similar spirit, Dummett claims that ‘to understand $A \vee B$, one need not understand $A \wedge B$ or $A \rightarrow B$’ (Dummett 1991, p. 223). If to understand a logical expression is to know its role in inference, and if the understanding of logical expressions is atomistic, then it is natural to assume that basic logical rules should be, in Dummett’s terminology, *pure*, i.e. such that exactly one logical operator figures in them.^8^

Let *orthodox inferentialism* be the view that the I- and E-rules of logical expressions must be harmonious and pure, and that any adequate axiomatisation of logic ought to be separable. The view can be traced back to Gentzen and has more recently been defended by Tennant in a number of writings (see e.g. Tennant 1997). Inferentialists such as Dummett and Prawitz relax the requirement of purity, and only require that basic logical rules be harmonious and that admissible axiomatisations of logic be separable. As Dummett puts it:

An impure $-introduction rule will make the understanding of $ depend on the prior understanding of the other logical constants figuring in the rule. Certainly we do not want such a relation of dependence to be cyclic; but there would be nothing in principle objectionable if we could so order the logical constants that the understanding of each depended only on the understanding of those preceding it in the ordering. (Dummett 1991, p. 257)

However, even relaxing the purity requirement in the way Dummett suggests, it is well known that harmony and separability alone are already incompatible with standard axiomatisations of classical logic.

## The Inferentialist Argument Against Classical Logic

Proof-theoretic constraints such as harmony and separability rule out Prior’s tonk. But, it may be argued, they rule out much more. For while the rules of intuitionistic logic are harmonious, standard formalizations of classical logic typically aren’t.^9^ For instance, the classical rule of double negation elimination 
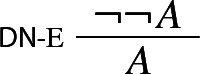
 is not in harmony with the standard rule of negation introduction: 
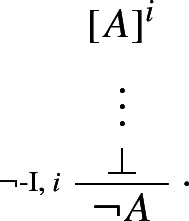
 The harmonious rule of negation elimination is the following *intuitionistic* rule: 
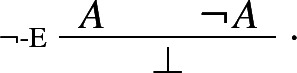
 Negation elimination, unlike its classical counterpart, allows one to infer from $\lnot A$ precisely what was required to assert $\lnot A$: a derivation of $\bot$ from *A*. It is easy to show that the rule is harmonious in the sense of satisfying both intrinsic harmony and local completeness.

### Example 3

(*Intuitionistic negation*) 
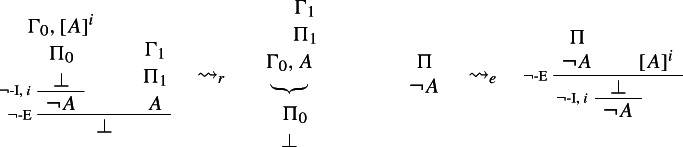


By contrast, the classical rule of double negation elimination is left, so to speak, in the cold. The same goes for any other classical rule, such as e.g. classical *reductio* or the Law of Excluded Middle: 
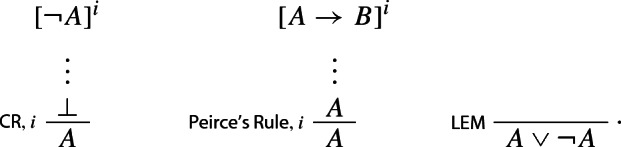
 Classical negation appears to be not harmonious.

It might be thought that the problem can be solved by simply supplying an extra set of harmonious I- and E-rules for one of the classical connectives, such as e.g. negation: 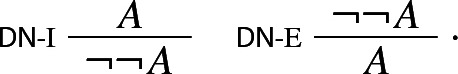 In this spirit, Weir (1986) proposes the following rules for disjunction: 
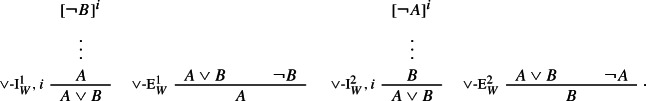
 The rules are pairwise harmonious, but they do not collectively satisfy intrinsic harmony, as the following derivation shows (see Weir 1986, pp. 476–478): 
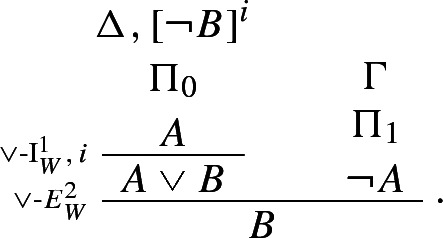
 Here there is no way one can in general derive *B* from a derivation of *A* from $\lnot B$, without appealing to Weir’s rules for disjunction.

Weir’s rules allow one to prove $A \vee \lnot A$ by means of an argument ending by just one application of disjunction introduction (Weir 1986, p. 469): 
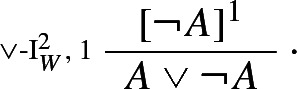
 The rule of double negation elimination is derived as follows: 
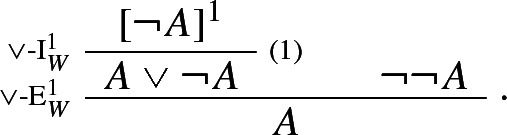
 However, it is easy to see that the idea of defining a single logical operator by means of multiple sets of harmonious introduction and elimination rules doesn’t work.^10^ For consider the following seemingly innocuous rules: 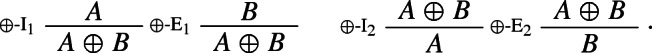 If they are taken to define a *single* connective, they validate Prior’s rules for tonk: 
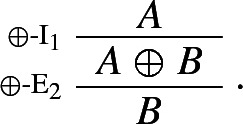
 In effect, Weir’s rules could be regarded as defining two harmless, and indeed harmonious, connectives $\vee '$ and $\vee ''$, one governed by $\vee$-I$^1_W$ and $\vee$-E$^1_W$ and one governed by $\vee$-I$^2_W$ and $\vee$-E$^2_W$, but neither of the two being equivalent to classical disjunction. In Sect. 3, I introduce genuinely harmonious classical rules for $\vee$.

Similarly, standard axiomatisations of classical logic are not separable. For instance, some uses of $\rightarrow$ such as Peirce’s Law, that $((A \rightarrow B) \rightarrow A) \rightarrow A$, are only derivable by means of rules for *both*$\rightarrow$ and $\lnot$. Intuitionists such as Dummett, Prawitz and Tennant have taken the lack of harmony and separability of standard axiomatisations of classical logic to show that classical rules such as double negation elimination are not logical (or that they are in some other sense defective), and that the logical rules we should adopt are those of *intuitionistic logic*, i.e. classical logic without the Law of Excluded Middle, double negation elimination and other equivalent rules [or perhaps of a weaker logic still (Tennant 1987, 1997)].^11^

However, while it is true that standard axiomatisations of classical logic are not harmonious, a number of non-standard axiomatisations of classical logic are both harmonious and separable. In particular, classical logic can be shown to be as proof-theoretically respectable as intuitionistic logic provided rules are given both for asserting and for *denying* complex statements (Rumfitt 2000; Incurvati and Smith 2010), where denial is taken to be a primitive speech act distinct from the assertion of a negated sentence (Parsons 1984; Smiley 1996). The resulting axiomatisation of classical logic is compatible with the orthodox inferentialist’s strictures (Rumfitt 2000). In particular, the rules for classical negation are as harmonious as the intuitionistic ones: they allow one to deny $\lnot A$ given the assertion of *A* and *vice versa*, and to deny *A* given the assertion of $\lnot A$ and *vice versa*. Alternatively, harmonious, pure, and separable axiomatisations of classical logic can be given once *multiple conclusions* are allowed (Read 2000; Cook 2005), either in a natural deduction or in a sequent-calculus setting.^12^

Inferentialists typically dismiss both of these moves. For one thing, it is unclear whether denial really is on a par with assertion. On the face of it, our linguistic practice appears to be assertion-based, as opposed to assertion-and-denial-based. For another, while it is possible to make sense of multiple-conclusion calculi, it would also seem that our inferential practice features arguments for at most *one* conclusion (Rumfitt 2008; Steinberger 2011c). As Ian Rumfitt puts it:

The rarity, to the point of extinction, of naturally occurring multiple-conclusion arguments has always been the reason why mainstream logicians have dismissed multiple-conclusion logic as little more than a curiosity. (Rumfitt 2008, p. 79)

While by no means decisive, these simple considerations make it worthwhile to ask whether an axiomatisation of classical logic that is both assertion-based and single-conclusion can be made consistent with the requirements of harmony, purity, and separability. The next four sections argue that it can, provided absurdity is interpreted as a punctuation sign and we allow for higher-level rules. New rules for disjunction will further make the axiomatisation to be presented in Sect. 6 compatible with Dummett’s Fundamental Assumption. I consider classical disjunction first (Sect. 3), before turning to absurdity (Sect. 4) and higher-level rules (Sect. 5).

## Classical Disjunction

From a classical inferentialist perspective, the standard rules for disjunction can be seen as unsatisfactory for at least two reasons.

To begin with, if the logic is classical, the standard introduction rules for $\vee$ are guaranteed not to respect Dummett’s Fundamental Assumption that, if one can introduce a complex statement, one could in principle introduce it by means of an argument ending with an application of one of the introduction rules for its main logical operator. The classical Law of Excluded Middle is a case in point: since in the present state of information it is not the case that, for every statement *A*, we can assert either *A* or its negation, we cannot introduce $A \vee \lnot A$ by means of an argument ending with an application of disjunction introduction, as the Fundamental Assumption requires.

Second, and relatedly, one often hears that the standard introduction rules for disjunction do not actually represent the way disjunctions are asserted in everyday practice, and that the meaning of ‘or’ in ordinary language is radically different from its meaning in logic. The complaint seems reasonable enough: we typically assert $A \vee B$ on the grounds that *A* and *B* cannot both be false—not because we already know that one of the disjuncts is true. As Scott Soames puts it:

nearly always when we assert the disjunction of *A* and *B* in ordinary language, we do so *not* because we already know that *A* is true, or because we already know that *B* is true. Rather, we assert the disjunction because we have some reason for thinking that it is highly unlikely, perhaps even impossible, that both *A* and *B* will fail to be true. (Soames 2003, p. 207)

This suggests the following new rules for disjunction:^13^
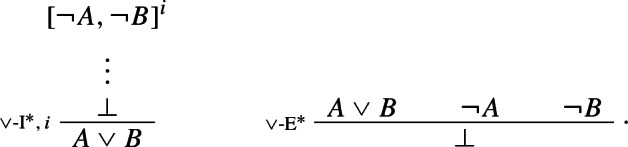
 Here the discharge of $\lnot A$ and $\lnot B$ might be vacuous, i.e. one does not need to actually use, and discharge, both of $\lnot A$ and $\lnot B$ in order to infer $A \vee B$ by one step of $\vee$-I$^*$. Thus for instance, 
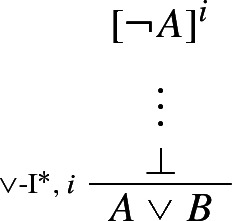
 counts as a legitimate application of $\vee$-I$^*$ This in turn highlights $\vee$-I$^*$’s *classicality*: what in textbook natural deduction systems would be an application of classical *reductio* (CR) immediately followed by one step of the standard rule of $\vee$-I is here turned into a single primitive step.^14^

The above rules are obviously harmonious: the elimination rule allows one to introduce precisely what was required to introduce $A \vee B$ in the first place, viz. a derivation of $\bot$ from $\lnot A$ and $\lnot B$. More precisely, the reduction step is as follows (where, since $\vee$-I$^*$ can discharge assumptions vacuously, only one of $\mathcal {D}_2$ and $\mathcal {D}_3$ might be present):

### Definition 4

($$\vee $$-*reduction*) 
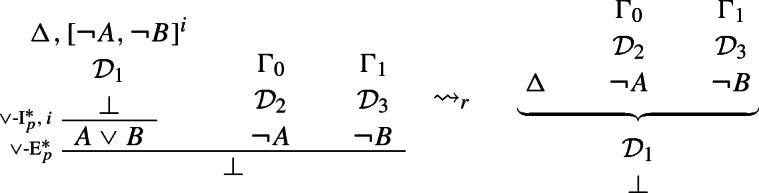


And here is the corresponding expansion step:

### Definition 5

($$\vee $$-*expansion*) 
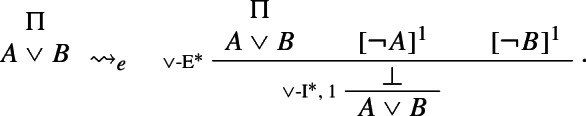


With these rules in place, the Law of Excluded Middle is provable on no assumptions via an argument ending with an application of $\vee$-I$^*$, as required by the Fundamental Assumption; one only needs to assume $\lnot A$ and $\lnot \lnot A$: 
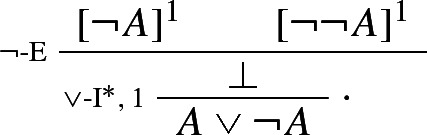
 The standard rules for disjunction and the new ones are interderivable given classical *reductio* or some equivalent rule such as double negation elimination. The standard two-part rule $\vee$-I can be derived using the new rule $\vee$-I$^*$ as follows: 
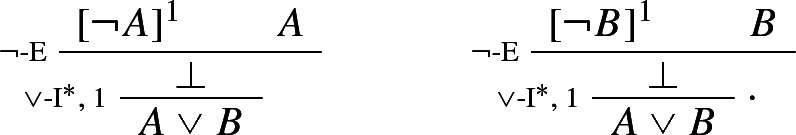
 As for the standard rule $\vee$-E, it can be derived using classical *reductio* and the new rule $\vee$-E$^*$: 
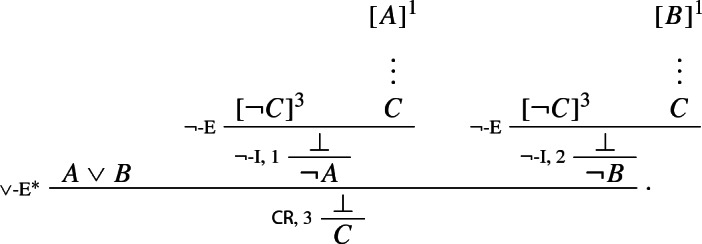
 Conversely, the new rule $\vee$-I$^*$ can be derived using CR from the standard two-part rule $\vee$-I, as follows: 
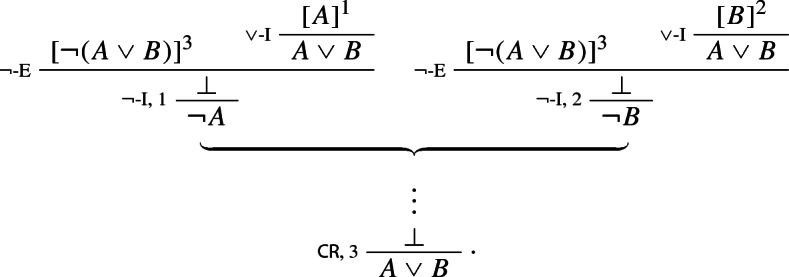
 Likewise, the new rule $\vee$-E$^*$ is derivable from the standard rule $\vee$-E: 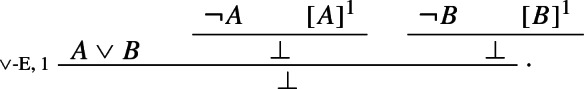


Classical though they may be, $\vee$-I$^*$ and $\vee$-E$^*$ do not suffice to yield a proof-theoretically acceptable axiomatisation of classical logic. For one thing, although they allow one to derive the Law of Excluded Middle, they do not yield either double negation elimination or *classical reductio*. And, absent double negation elimination (or some equivalent rule, such as classical *reductio*), they do not even yield the standard rule of disjunction elimination. For another, the revised rules are impure, since more than one logical operator figures in their schematic form. They are therefore unacceptable by orthodox inferentialist standards.

Both problems can be solved, provided that classical logicians interpret absurdity as a *logical punctuation sign* and are willing to allow for *higher-level rules* in their formalisation of logic. The next two sections introduce these two ingredients in turn.

## Absurdity as a Punctuation Sign

It is notoriously difficult to offer an adequate inferentialist account of absurdity. Dag Prawitz suggests that $\bot$ be defined by the *empty* I- rule. That is, in his view, there is no canonical way of introducing $\bot$. He writes:

the introduction rule for $\bot$ is empty, i.e. it is the rule that says that there is no introduction whose conclusion is $\bot$. (Prawitz 2005, p. 685)

In Prawitz’s view, the rule can be shown to be in harmony with *ex falso quodlibet*^15^: 
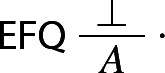
 On the other hand, Dummett has claimed that $\perp$ should rather be defined by the following infinitary rule of $\perp$-introduction 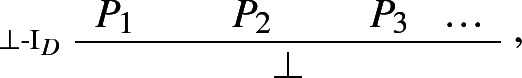 where the $P_n$ are all the atoms of the language, which Dummett takes to be jointly inconsistent (see Dummett 1991, pp. 295–256). The idea is to specify canonical grounds for $\bot$ that can never obtain: no rich enough language will allow for the possibility in which all atoms, including basic contraries such as ‘This table is all red’ and ‘This table is all white’, can be proved—or so the thought goes. The rule is evidently harmonious with EFQ: one can derive from an assertion of $\bot$ precisely what was required for asserting $\bot$ in the first place.

Both Prawitz’s and Dummett’s accounts are problematic, however. Dummett’s rule is non recursive and makes the meaning of $\bot$ dependent on the expressiveness of one’s language. After all, it may be argued that atoms need not be in general incompatible. As for Prawitz’s account of $\bot$, the very thought that $\bot$ has content makes the meaning of negation dependent on the meaning of absurdity, and hence violates the orthodox inferentialist’s demand for purity.

An alternative, and more promising, proposal views $\bot$ as a *logical punctuation sign* (Tennant 1999; Rumfitt 2000). Thus, Tennant writes that

an occurrence of ‘$\bot$’ is appropriate only within a proof [...] as a kind of structural punctuation mark. It tells us where a story being spun out gets tied up in a particular kind of knot—the knot of a patent absurdity, or self contradiction. (Tennant 1999, p. 204)

Similarly, Rumfitt suggests that $\bot$ ‘marks the point where the supposition [...] has been shown to lead to a logical dead end, and is thus discharged, prior to an assertion of its negation’ (Rumfitt 2000, pp. 793–794). On such a view, $\mathsf {EFQ}$ becomes a *structural rule*, i.e. a form of weakening on the right (Steinberger 2009a, 2011b).

Formally, to treat $\bot$ as a logical punctuation sign is to switch from a set-formula framework (SET-FMLA), i.e. a framework in which the premises of an argument form a set and its conclusion is always a singleton, to a to a set-formula-or-empty-set framework (SET-SET$_\emptyset$), i.e. a framework in which the premises of an argument form a set and its conclusion is always either a singleton or the empty set. Clearly, both options are compatible with the orthodox inferentialist’s rejection of multiple-conclusions.^16^ In the remainder of this paper, I will treat $\bot$ as a logical punctuation sign.^17^

## Higher-Level Rules

Now to higher-level rules. Natural deduction systems involve rules, such as arrow introduction, which allow one to discharge *assumptions*. But what exactly is an assumption? Schroeder-Heister (1984) suggests that to assume some formulae $\beta _1, \ldots , \beta _n$ is technically just to treat these formulae as *temporary axioms*:

Assumptions in sentential calculi technically work like additional axioms. A formula $\alpha$ is derivable from formulas $\beta _1, \ldots , \beta _n$ in a calculus $\mathcal {C}$ if $\alpha$ is derivable in the calculus $\mathcal {C}'$ resulting from $\mathcal {C}$ by adding $\beta _1, \ldots , \beta _n$ as axioms. But whereas “genuine” axioms belong to the chosen framework and are usually assumed to be valid in some sense, assumptions bear an ad hoc character: they are considered only within the context of certain derivations. (Schroeder-Heister 1984, p. 1284)

But if assumptions just are ad hoc axioms, one should also be free to use ad hoc rules in the context of a derivation. Thus Schroeder-Heister again:

Instead of considering only ad hoc axioms (i.e. assumption formulas) we can also regard ad hoc inference rules, that is, inference rules [...] used as assumptions. Assumption rules technically work like additional basic rules: $\alpha$ is derivable from assumption formulas $\beta _1, \ldots , \beta _n$ and assumption rules $\rho _1, \ldots , \rho _m$, in $\mathcal {C}$ if $\alpha$ is derivable in $\mathcal {C}'$, where $\mathcal {C}'$ results from $\mathcal {C}$ by adding $\beta _1, \ldots , \beta _n$ as axioms and $\rho _1, \ldots , \rho _m$ as basic inference rules. (Schroeder-Heister 1984, p. 1285)

Armed with Tennant’s account of absurdity as a logical punctuation sign and with Schroeder-Heister’s higher-level rules, let us now turn to classical logic.

On the foregoing assumptions, *modus ponens* can be formulated as a higher-level rule, as follows: 
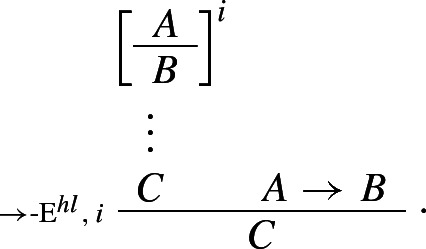
 The standard rule of arrow elimination is obtained by setting *C* equal to *B* (then, given a derivation of *A*, one may conclude *B* from  and *A*). Similarly, classical *reductio* can be rewritten as a *structural* rule, as follows: 
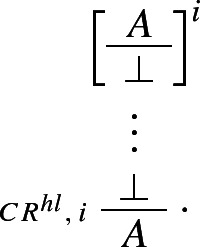
 If one can derive a contradiction from the assumption that *A* itself leads to a contradiction, one can discharge that assumption and infer *A*. The rule is structural since no logical operator figures in it: recall, following Tennant, we are interpreting $\bot$ as shorthand for the empty set, rather than as a propositional constant.^18^ Finally, our proposed *impure* rules for disjunction can now be presented as *pure* harmonious rules. The I-rule can be read as stating that, if one can derive absurdity from the rules  and  one may discharge the rules and infer $A \vee B$. More formally: 
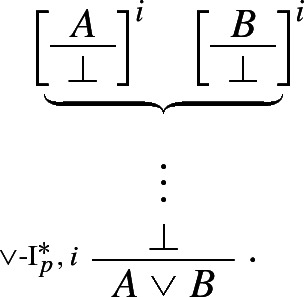
 Conversely, the corresponding E-rule states that, given a proof of $A \vee B$, one may infer $\bot$ from the rules  and : 
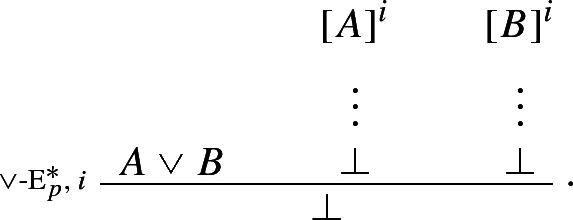
 It is easy to show that this pair of I- and E-rules is just as harmonious as its impure counterpart $\{\vee$-I$^*, \vee$-E$^*\}$.

The new rules $\vee$-$\hbox {I}_p^*$ and $\vee$-$\hbox {E}_p^*, \mathsf{CR}^{hl}$, and the standard I- and E-rules for conjunction, implication, and negation, together afford a harmonious and pure axiomatisation of classical propositional logic (henceforth, **CPL**), in which each of the connectives is treated as a primitive.^19^ Call this formalization **Ncp**.

In keeping with Schroeder-Heister’s original treatment of higher-level rules, **Ncp** only allows for the *assumption* of rules. However, once rules can be assumed, it is difficult to see why rules couldn’t also figure as *conclusions*. Consider the following structural rule, where depending on graphic convenience I sometimes write  as *A* / *B*: 
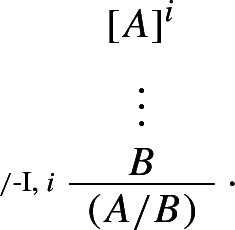
 The rule allows one to derive the rule *A* / *B* from a derivation of *B* from *A*, discharging *A*. The parentheses ensure unique readability: they indicate that the object , as opposed to simply *A*, follows from a derivation of *B* from *A*.^20^ The rule is naturally paired with the following, also purely structural, rule: 
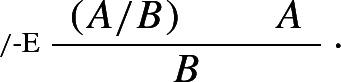
 This says that, given the rule , *B* can be derived given *A*.

The introduction and immediate elimination of  /  gives rise to what we may call a *maximum rule*, i.e. a rule occurrence that is both the consequence of an application of /-I and the major premise of an application of /-E. Unsurprisingly, maximum rules can be ‘levelled’, as shown by the following reduction:

### Definition 6

( / -*reduction*) 
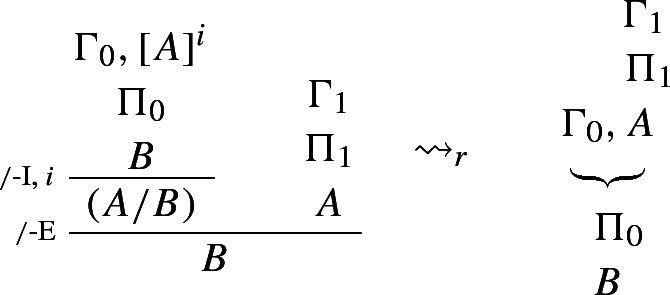


The definition of intrinsic harmony given in Sect. 1 can be generalised accordingly, as the possibility of eliminating maximum formulae *and rules*.

Although they bear a close resemblance to $\rightarrow$-I and $\rightarrow$-E, the *structural* rules  / -I and  / -E should be sharply distinguished from the *operational* rules $\rightarrow$-I and $\rightarrow$-E: while $\rightarrow$-I and $\rightarrow$-E allow one to respectively introduce and eliminate an *operator*,  / -I and  / -E allow one to respectively introduce and eliminate a *rule*.

It might be insisted that  / -I and  / -E are just $\rightarrow$-I and $\rightarrow$-E in disguise. However, the objection would miss the point: from the fact that  could be interpreted as $A \rightarrow B$, it doesn’t follow that it *is* to be so interpreted. An analogy helps to illustrate the point. Consider a bilateralist setting, where $+$ and − are force signs, $+A$ and $-A$ are to be respectively read as ‘*A*? Yes’ and ‘*A*? No’, the assumption of $+A$ is to be interpreted as ‘*A*? Suppose yes’, and $\bot$ is interpreted as the empty set. Now consider the following bilateralist form of indirect proof: 
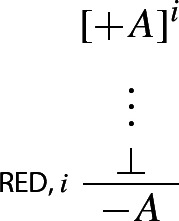
 Since $+$ and − are force signs that don’t affect propositional content, RED is effectively a structural rule that, in a bilateralist framework, allows one to deny *A* given a derivation of $\bot$ from the assumption $+A$. It could be objected that RED is a form of negation introduction in disguise (Murzi and Hjortland 2009, p. 486). But the point would not be well taken. For while the denial force sign in RED could be interpreted as an external negation, it doesn’t follow from this that it *is* be so interpreted (Incurvati and Smith 2010, pp. 9–10).

Now let $\mathbf{Ncp }^{+}$ be the result of closing **Ncp** under  / -I and  / -E. To give the reader a feel of the new system, we prove two classical principles. We first prove the Excluded Middle:

### Example 7

(*Excluded middle*) 
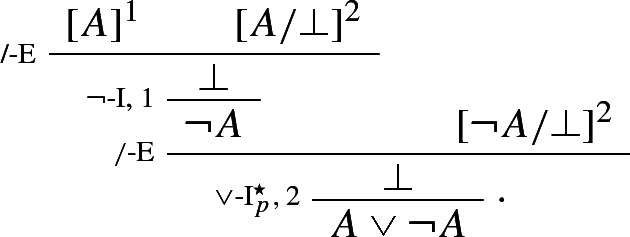


We then prove Peirce’s Law in rule form, only making use of rules for $\rightarrow$ (and structural rules):

### Example 8

(*Peirce’s rule*) 
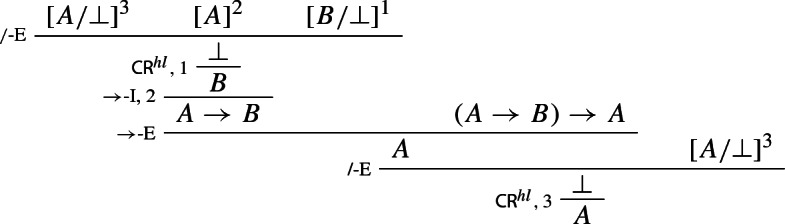


The next section shows that $\mathbf{Ncp }^{+}$ is not only harmonious, but also satisfies the more demanding requirement of separability.

## Normalization for $\mathbf{Ncp }^{+}$

Following (and generalising) Prawitz (1965), we prove normalization and subformula property theorems for $\mathbf{Ncp }^{+}$. The subformula property theorem entails the separability property as an immediate corollary. First, we define $\mathbf{Ncp }^{+}$.

### Definition 9

Formulae of $\mathbf{Ncp }^{+}$ are built up from atoms and from the standard binary connectives $\wedge , \vee , \rightarrow$, and the unary connective $\lnot$. Absurdity ($\bot$) is a logical ‘punctuation sign’, and hence not an atom. The rules for $\wedge , \rightarrow$, and $\lnot$ are the standard ones: $\wedge$-I, $\wedge$-E, $\rightarrow$-I, $\rightarrow$-E, $\lnot$-I, $\lnot$-E. The rules for $\vee$ are non-standard: $\vee$-I$^*_p$ and $\vee$-E$^*_p$. There are three structural rules: $\mathsf {CR}^{hl}, /$-I, and  / -E.

### Definition 10

Objects of $\mathbf{Ncp }^{+}$ are divided into *levels*. Atomic formulae and compound formulae of the form $\lnot A, A \wedge B, A \vee B$, and $A\rightarrow B$ are of level 0. Rules of the form *A* / *B* are of level 1. Rules of the form  or  are of level 2. And so on.

I use Greek letters $\gamma , \delta$ (possibly with subscripts) as metavariables ranging over formula occurrences, occurrences of $\bot$, and rule occurrences. We then prove in three easy steps that $\mathbf{Ncp }^{+}$ really gives us classical propositional logic.

### Fact 11

The operational rules of $\mathbf{Ncp }^{+}$ are pure.

### Lemma 12

The standard disjunction rules $\vee$-I and $\vee$-E are interderivable with $\vee$-I$^*_p$ and $\vee$-E$^*_p$, given $\mathsf {CR}$.

*Proof*: left as an exercise to the reader (the proof is essentially already given in Sect. 3).

### Lemma 13

$\mathsf{{CR}}^{hl}$ and $\mathsf{{CR}}$ are interderivable in minimal logic.

*Proof*: It is enough to observe that $\lnot A$ and  are interderivable. We first prove that  follows from $\lnot A$: 
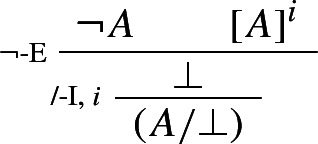

We then prove the converse implication: 
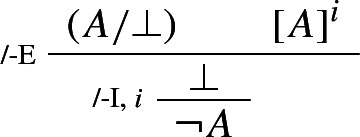

### Corollary 14

$\mathbf{Ncp }^{+}$ is a sound and complete axiomatisation of **CPL**.

*Proof*: this follows from Lemmas 12 and 13, given the observation that minimal logic together with $\mathsf{CR}$ yields a sound and complete axiomatisation of **CPL**.

Next, we define the notions of *maximum rule*, *local peak*, *normal deduction*, and *subformula*:

### Definition 15

(*Maximum formula*) A *maximum formula* in $\Pi$ is a formula occurrence in $\Pi$ that is the consequence of an application of an I- rule or a $\bot$-rule (namely, CR, CR$^{hl}$, or EFQ) and the major premise of an E-rule.

### Definition 16

(*Maximum rule*) A *maximum rule* in $\Pi$ is a rule occurrence in $\Pi$ that is the consequence of an application of an I- rule and the major premise of an E-rule.

### Definition 17

(*Local peak*) A *local peak* in $\Pi$ is either a maximum formula or a maximum rule in $\Pi$.

### Definition 18

(*Normal deduction*) A normal deduction is a deduction which contains no local peaks.

### Definition 19

(*Subformula*) The notion of a subformula in $\mathbf{Ncp }^{+}$ is inductively defined by the following clauses:

1. *A* is a subformula of *A*;
2. *A* is a subformula of $\lnot A$;
3. *A* and *B* are subformulae of *A* / *B*;
4. If $B \wedge C, B \vee C$, or $B \rightarrow C$ is a subformula of $\gamma$ (where $\gamma$ may be a formula or a rule), then so are *B* and *C*.

We can now prove that every deduction in $\mathbf{Ncp }^{+}$ converts into a normal deduction. To this end, we first need to show that local peaks can always be removed.

Let $\Pi$ be a derivation of *E* from $\Gamma$ that contains a local peak $\gamma$ that is a consequence of an application of an I-rule and major premise of an application of an E-rule. Then, following Prawitz (1965, p. 36), we say that $\Pi '$ is a *reduction of*$\Pi$*at*$\gamma$ if $\Pi '$ is obtained from $\Pi$ by removing $\gamma$ by an application of a *reduction procedure*. The reduction for our modified disjunction rules is as follows

### Definition 20

($$\vee $$-*reduction*) 
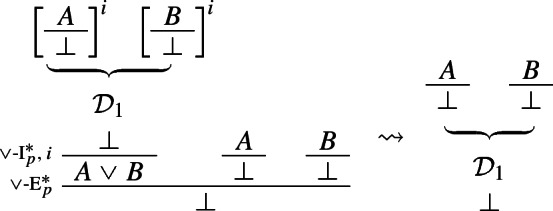


The reduction for / has been introduced in Definition 6. The remaining conversion steps are standard (see Prawitz 1965, Chapter 2).

In our next step, we prove that we can restrict applications of $\mathsf {CR}^{hl}$ to the case where its conclusion is atomic.

### Theorem 21

$(\mathsf{{CR}}^{hl}$-restriction) Applications of $\mathsf{{CR}}^{hl}$ can always be restricted to the case where the conclusion is atomic.

### Proof

We generalise Prawitz’s original proof (Prawitz 1965, pp. 39–40) to the present case involving our higher-level rules for disjunction and the higher-level structural rule $\mathsf {CR}^{hl}$. Let $\Pi$ be a deduction in $\mathbf{Ncp }^{+}$ of *A* from $\Gamma$ in which the highest degree of a consequence of an application $\alpha$ of $\mathsf {CR}^{hl}$ is *d*, where $d > 0$ and the *degree* of a formula *A* is defined as the number of occurrences of logical operators in *A* (see Prawitz 1965, p. 16). Let *F* be a consequence of an application $\alpha$ of $\mathsf {CR}^{hl}$ in $\Pi$ such that its degree is *d* but no consequence of an application of $\mathsf {CR}^{hl}$ in $\Pi$ that stands above *F* is of degree greater than or equal to *d*. Then $\Pi$ has the form 
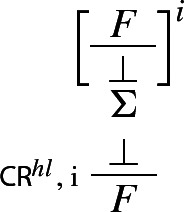
 where  is the set of derivations discharged by $\alpha$, and *F* has one of the following forms: $\lnot A, A \wedge B, A \rightarrow B$, or $A \vee B$.^21^ In the respective cases, we transform $\Pi$ into derivations which either do not contain applications of $\mathsf {CR}^{hl}$ or have consequences of applications of $\mathsf {CR}^{hl}$ of degree less than *d*. Here are the transformations for negation 
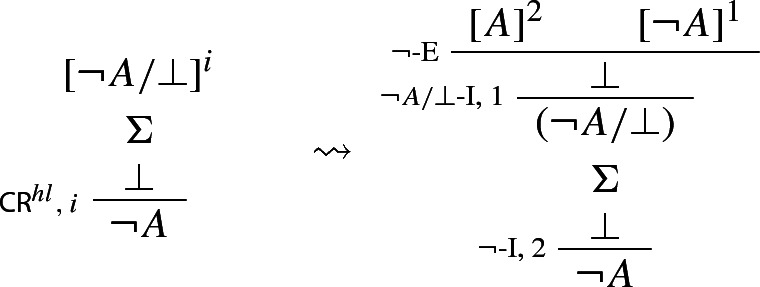
 conjunction 
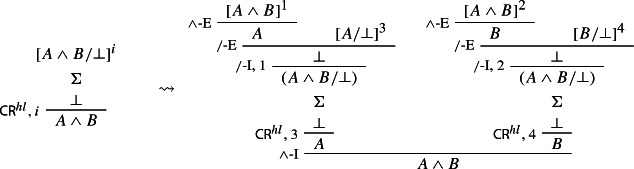
 and the conditional 
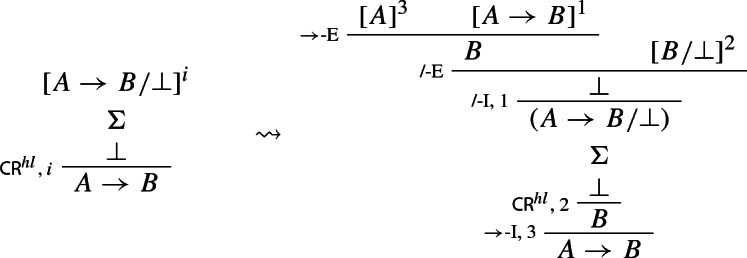


The case for disjunction can be dealt with similarly as follows: 
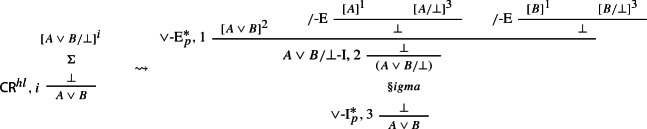
 The new applications of $\mathsf {CR}^{hl}$ (if any) have consequences of degrees less than *d*. Hence, by successive applications of the above procedures we finally obtain a deduction of *A* from $\Gamma$ in which every consequence of every application of $\mathsf {CR}^{hl}$ is atomic. $\square$

We now generalise Prawitz’s proof that his axiomatisation of **CPL** is normalisable. We begin with some definitions, largely following Prawitz (1965, p. 25 and ff; and p. 41).

### Definition 22

(*Top and end formulae*) A *top-formula* in a formula-tree $\Pi$ is a formula-occurrence or an occurrence of $\bot$ that does not stand immediately below any formula occurrence or occurrence of $\bot$ in $\Pi$. An *end-formula* in a formula-tree $\Pi$ is a formula-occurrence or occurrence of $\bot$ that does not stand immediately above any formula occurrence or occurrence of $\bot$ in $\Pi$.

### Definition 23

(*Top and end rules*) A *top-rule* in a formula-tree $\Pi$ is a rule-occurrence that does not stand immediately below any formula occurrence in $\Pi$ or occurrence of $\bot$. An *end-rule* in a formula-tree $\Pi$ is a rule-occurrence that does not stand immediately above any formula occurrence or occurrence of $\bot$ in $\Pi$.

### Definition 24

(*Thread*) A sequence $\gamma _1, \gamma _2, \ldots , \gamma _n$ of formula occurrences, or occurrences of $\bot$, or rule occurrences, in a formula-tree $\Pi$ is a *thread* in $\Pi$ if (i) $\gamma _1$ is a top-formula or a top-rule in $\Pi$, (2) $\gamma _i$ stands immediately above $\gamma _{i + 1}$ in $\Pi$ for each $i < n$, and (3) $\gamma _n$ is the end-formula or end-rule of $\Pi$. We say that $\gamma _i$ stands *above* (*below*) $\gamma _j$ if $i < j (i > j)$.

### Definition 25

(*Subtree*) If $\delta$ is a formula occurrence, or occurrence of $\bot$, or rule occurrence, in the tree $\Pi$, the *subtree of*$\Pi$*determined by*$\gamma$ is the tree obtained from $\Pi$ by removing all formula occurrences or occurrences of $\bot$ except those in $\gamma$ and the ones above $\gamma$.

### Definition 26

(*Side-connectedness*) Let $\gamma$ be a formula occurrence, or occurrence of $\bot$, or rule occurrence in $\Pi$, let $(\Pi _1, \Pi _2, \ldots , \Pi _n/\gamma )$ be the subtree of $\Pi$ determined by $\gamma$ and let $\gamma _1, \gamma _2, \ldots , \gamma _n$ be the end-formulae or end-rules of, respectively, $\Pi _1, \Pi _2, \ldots , \Pi _n$. We then say that $\gamma _i$ is *side-connected* with $\gamma _j$, for $i, j \le n$

### Definition 27

(*Branches*) A *branch* in a deduction is the initial part $\gamma _1, \gamma _2, \ldots , \gamma _n$ of a thread in the deduction such that either (i) $\gamma _n$ is the first formula occurrence in the thread that is the minor premise of an application of either $\rightarrow$-E or $\lnot$-E, or the formula occurrence or occurrence of $\bot$ in the thread that is the minor premise of  / -E or a minor premise of $\vee$-$\hbox {E}_{p}$; or (ii) $\gamma _n$ is the last formula occurrence of the thread (i.e. the end formula of the deduction) if there is no such minor premise in the thread. A branch that is also a thread that thus contains no minor premise of $\rightarrow$-E, $\lnot$-E, or  / -E, or $\vee$-$\hbox {E}_{p}$ is a *main branch*.

### Theorem 28

(Normalization) If $\Gamma \vdash _{\mathbf{Ncp }^{+}} \gamma$, then there is a normal deduction in $\mathbf{Ncp }^{+}$ of $\gamma$ from $\Gamma$ (where $\Gamma$ is a possibly empty set of formulae or rules).

### Proof

Let $\Pi$ be a deduction in $\mathbf{Ncp }^{+}$ of $\gamma$ that is as described in Theorem 21. Let the degree of rule *R* be the number of occurrences of logical operators in *R* (recall, $\bot$ is not a logical operator). Now let $\delta$ be a local peak in $\Pi$ such that there is no other local peak in $\Pi$ of higher degree than that of $\delta$ and such that local peaks in $\Pi$ that stand above a formula occurrence side-connected with $\delta$ (if any) have lower degrees than $\delta$. Let $\Pi '$ be a reduction of $\Pi$ at $\delta$. The new local peaks that may arise from this reduction are all of lower degrees than that of $\delta$. Moreover, $\Pi '$ is still as described above. Hence, by a finite number of reductions, we obtain a normal deduction of $\gamma$ from $\Gamma$.^22^$\square$

### Theorem 29

Let $\Pi$ be a normal deduction in $\mathbf{Ncp }^{+}$, and let $\beta = \gamma _1, \gamma _2, \ldots , \gamma _n$ be a branch in $\Pi$. Then, there is a formula occurrence, or occurrence of $\bot$, or rule occurrence $\gamma _i$, called the *local valley* in $\beta$, which separates two (possibly empty) parts of $\beta$, respectively called the *E-* and *I-part* of $\beta$, with the properties:

1. Each formula or rule occurrence $\gamma _j$ in the E-part (i.e. $j < i$) is a major premise of an E-rule and contains $\gamma _{j + 1}$ as a subformula.
2. $\gamma _i$, provided $i \not =n$, is a premise of an I-rule or of CR$^{hl}$.
3. Each formula $\gamma _j$ in the I-part except the last one (i.e. $i< j < n$) is a premise of an I-rule and is a subformula of $\gamma _{j + 1}$.

### Proof

The formula or rule occurrences in $$\beta $$ that are major premises of E-rules precede all formula occurrences or occurrences of $$\bot $$ in $$\beta $$ that are premises of I-rules or of CR$$^{hl}$$. Otherwise, there would be a first formula or rule occurrence in $$\beta $$ which is a major premise of an E-rule but succeeds a premiss of an I-rule or of CR$$^{hl}$$, and such a formula or rule occurrence would be a local peak, contrary to the assumption that $$\Pi $$ is normal. Now let $$\gamma _i$$ be the first formula occurrence or occurrence of $$\bot $$ in $$\beta $$ that is premise of an I-rule or of CR$$^{hl}$$ or, if there is no such segment, let $$\gamma _i$$ be $$\gamma _n$$. Then, $$\gamma _i$$ is a local valley as described in the theorem. Obviously, $$\gamma _i$$ satisfies both 1. and 2. Moreover, every formula occurrence or occurrence of $$\bot $$$$\gamma _j$$ such that $$i< j < n$$ is a premise of an I-rule or of CR$$^{hl}$$. However, the latter possibility is excluded, since a premise of CR$$^{hl}$$ is an occurrence of $$\bot $$ and can be consequence of an E-rule only. Hence, 3. is also satisfied. $$\square $$

### Corollary 30

(Subformula property) Each formula occurring in a normal deduction $\Pi$ of $\gamma$ from $\Gamma$ is a subformula of $\gamma$ or of one of the formulae in $\Gamma$.

Prawitz (1965, pp. 42–43) proves this result for his own formalization of **CPL**, which includes the rules for $\wedge , \rightarrow$, and $\mathsf{C}\!\mathsf{R}$, and where $\lnot A$ is defined as $A \rightarrow \bot$. In Prawitz’s system, the theorem holds for every formula in $\Pi$, ‘except for assumptions discharged by applications of $\mathsf{C}\!\mathsf{R}$ and for occurrences of $\bot$ that stand immediately below such assumptions’. Prawitz’s proof carries over to $\mathbf{Ncp }^{+}$, this time without exceptions. Informally, this can be shown by considering, in the new $\mathbf{Ncp }^{+}$ setting, the exceptions to Prawitz’s original theorem, viz. that (i) assumptions discharged by applications of $\mathsf{C}\!\mathsf{R}$ and (ii) occurrences of $\bot$ that stand immediately below such assumptions may not be subformulae of either $\gamma$ or some of the formulae in $\Gamma$. Concerning (i), we then notice that it is a consequence of Prawitz’s theorem that, if $B/\bot$ is an assumption discharged by $\mathsf{C}\!\mathsf{R}^{hl}$ in a normal deduction of *A* from $\Gamma$, then *B* is a subformula of *A* or of some subformula of $\Gamma$. As for (ii), the problem disappears as soon as we treat $\bot$ as a logical punctuation sign. For a fuller proof, we first *order branches* according to the following definition, still following and generalising Prawitz’s original proof.

### Definition 31

(*Ordering of branches*) A main branch (i.e. a branch that ends with an end-formula of $\Pi$) has order 0. A branch that ends with a minor premise of an application of $\rightarrow$-E, /-E, $\lnot$-E, or $\vee$-$\hbox {E}_p$ is of order $n + 1$ if the major premise of this application has order *n*.

We now prove Corollary 30 by induction on the order of branches.

### Proof

Let $\Pi$ be a normal deduction in $\mathbf{Ncp }^{+}$. We show that the corollary holds for all formula occurrences or occurrences of $\bot$ in a branch of order *p* if it holds for formula occurrences in branches of order less than *p*. Let $\beta$ be $\gamma _1, \gamma _2, \ldots , \gamma _n$ and let $\gamma _i$ be the local valley of $\beta$. For $\gamma _n$ the assertion is immediate: either $\gamma _n = \gamma$, or $\gamma _n$ is a minor premise of an application of $\rightarrow$-E, $\lnot$-E, $\vee$-$\hbox {E}_p$, or  / -E with a major premise of the form either $A \rightarrow B$, or $\lnot A$, or $A \vee B$, or *A* / *B* that belongs to a branch of order $p - 1$. Hence, by Theorem 29, the corollary holds for all $\gamma _j$ such that $i< j < n$. If $\gamma _1$ is not discharged by an application of CR$^{hl}$ or $\vee$-$\hbox {I}_p$, then either $\gamma \in \Gamma$ or $\gamma _1$ is a formula $A_1$ discharged by an application $\alpha$ of either $\rightarrow$-I, $\lnot$-I, or  / -I such that the consequence of $\alpha$ has the form either $A_1 \rightarrow B$, or $\lnot A_1$, or $A_1/B$ and belongs to the I-part of $\beta$ or to some branch of order less than *p*. Hence, in this case, $A_1$ is a subformula of the required kind, and, by Theorem 29, the same holds for all $A_j$ such that $j \le i$. Finally, if $\gamma _1$ is a rule discharged by an application of CR$^{hl}$ or of $\vee$-$\hbox {I}_p$, then $\gamma _1$ is a minor premise of $\vee$-$\hbox {E}_p$, and so $\gamma _1 = \gamma _n$; hence, also in the latter three cases, the proof is complete. $\square$

### Theorem 32

(Separation property) Any normal deduction only consists of applications of the rules for the connectives occurring in the undischarged assumptions, if any, or in the conclusion, plus possibly $\mathsf{{CR}}^{hl}$.

### Proof

This follows at once from Corollary 30, by inspection of the inference rules. $\square$

## Objections and Replies

Recall, the intuitionist’s contention was that classical logic cannot be regimented in a proof-theoretically acceptable way: classical logic, the intuitionist complained, is bound to be inharmonious or inseparable. The foregoing formalization of classical logic, if acceptable at all, shows that this accusation is misplaced. $\mathbf{Ncp }^{+}$ provides a single-conclusion and assertion-based axiomatisation of **CPL** satisfying the orthodox inferentialist’s requirements of harmony, purity, and separability. The intuitionist’s error, classical inferentialists may diagnose, was to think that the extra deductive power enjoyed by negation, disjunction, and implication in classical logic had to be owed to their respective I- and E-rules. But, classical inferentialists may argue, this was a mistake: the extra deductive power essentially derives from a different (and richer) understanding of $\bot$.

Intuitionists might object that the foregoing axiomatisation of classical logic, if proof-theoretically kosher, is incompatible with inferentialism. Rumfitt has recently made the point. As he puts it:

A set/formula sequent represents an actual argument, in which a reasoner passes from a set of premises to a conclusion. Hence the correctness of such a sequent can be related to the intuitive acceptability of the corresponding inferential passage. Where a speaker fails to reach such a conclusion, however, we do not have an inference; we merely have a list of statements. Accordingly, we cannot explain the correctness of a set/formula-or-empty sequent directly in terms of the intuitive acceptability of an inference. (Rumfitt 2017, p. 237)

The argument fails to convince, though. Consider, for instance, the rule of negation elimination: 
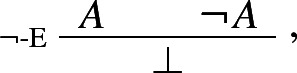
 where $\bot$ is interpreted as a logical punctuation sign, i.e. as the empty set. Then, the rule correctly represents a plausible pattern of inference: upon deriving both *A* and $\lnot A$, a rational agent *stops* her reasoning and examines instead which of the assumptions on which *A* and $\lnot A$ depend must be given up.

It may be insisted that, *qua* structural rule, $\mathsf {CR}^{hl}$, and hence classicality, has not been proof-theoretically justified. As Priest puts it:

[I]ntroduction and elimination rules are superimposed on structural inferential rules [...] and the question therefore arises as how *these* are to be justified. (Priest 2006, p. 179)

However, a parallel argument would show that intuitionistic logic cannot be fully proof-theoretically justified either, since intuitionistically valid structural principles such as (say) weakening and contraction do not appear to be justifiable by means of proof-theoretic requirements such as harmony and separability. The inferentialist requirements of harmony, purity, and separability pertain (and have always pertained) to logical operators, and it is consistent with these requirements that structural rules be justified, or criticised, in non-proof-theoretic ways.

Intuitionists might retort that, although this may well be true, classical logicians need *stronger* structural assumptions, which, they may add, still makes classical logic epistemically suspect. But all that follows from this is that the proper intuitionist challenge to the classical logician is not a proof-theoretic one. Rather, it must be directed to the classicist’s extra structural assumptions. More precisely, in the foregoing framework, the challenge should be directed to the classicist’s logic of absurdity. Stephen Read makes the point, albeit in a slightly different context:

The constructivist can still mount a challenge to classical logic. But we now see where that challenge should be concentrated—and where it is misguided. The proper challenge is to Bivalence, and to the classical willingness to assert disjunctions, neither of whose disjuncts is separately justified [...]. (Read 2000, pp. 151–152)

In the present framework, the challenge should be mounted to the inferentialist’s willingness to infer *A* if the assumption that *A* leads to a dead end (less figuratively: the rule ‘From *A*, infer $\bot$’) itself leads to a dead end (yields $\bot$).

## Conclusions

Dummett once wrote that the proof-theoretic deficiency of the classical rules for negation (in a standard SET-FMLA setting) is ‘a strong ground for suspicion that the supposed understanding [of classical negation] is spurious’ (Dummett 1991, p. 299). However, even conceding that the meaning and understanding of logical connectives are inexorably tied to the proof-theoretic demands of harmony, purity, and separability, Dummett’s conclusion is unwarranted: *pace* the intuitionist’s contention that classical logic is proof-theoretically defective, $\mathbf{Ncp }^{+}$ enjoys exactly the same proof-theoretic properties as the standard axiomatisations of intuitionistic logic. Moreover, the new rules for disjunction allow one to prove directly the Law of Excluded Middle, thus vindicating the inferentialist thought that ‘what is implicit in the totality of cases of the introduction-rule for a connective is that they exhaust the grounds for assertion of that specific conclusion’ (Read 2008, p. 289). Classical logic may well be shown to be defective—for instance, on the grounds that it is incompatible with an anti-realist metaphysics (see e.g. Wright 1992, Chapter 2), or that it does not accommodate the semantic and soritical paradoxes (see e.g. Field 2008). But, even assuming an orthodox inferentialist account of logical expressions, the grounds for restricting certain classical principles are not proof-theoretic.

## Footnotes

1 I should stress at the outset that, even though the axiomatisation is novel, some of its main ingredients have been present in the literature for some time. For one thing, the De Morgan-like rules for disjunction to be introduced in Sect. 3 are already briefly discussed in Murzi (2010 Ch. 7, §4.12), Murzi and Steinberger (2013, p. 181, fn. 37), and, more recently, Prawitz (2015, p. 29) and Pereira and Rodriguez (2017, p. 1156). For another, the interpretation of classical *reductio* as a structural rule offered in Sect. 5 can already be found, in essence, in Schroeder-Heister’s dissertation, only available in German (see Schroeder-Heister 1981, §18, *Absurdität als Grundbegriff*, p. 241 and ff). Among other things, Schroeder-Heister also indicates how to modify his normalisation theorem for classical logic in order for it to apply to an axiomatisation of classical logic in which classical *reductio* is structural. The ideas and results presented in this paper were found independently of Schroeder-Heister, and the proof strategies adopted for proving the normalisation and separability results of Sect. 6 are imported from Prawitz rather than Schroeder-Heister. I am grateful to an anonymous reviewer for bringing his dissertation to my attention in February 2018.

2 See Popper (1947, p. 220), Kneale (1956, pp. 254–255), and Dummett (1991, p. 247).

3 We will give a precise and slightly more general definition of a maximum formula in Sect. 6 below.

4 For instance, a connective $\odot$ satisfying the standard I-rules for $\wedge$ but only one of its E-rules would be intrinsically harmonious, and yet intuitively disharmonious: its E-rule would not allow one to infer from $A \odot B$ all that was required to introduce $A \odot B$ in the first place.

5 For an overview see Steinberger (2011b). Tennant’s conception of harmony is further discussed in Steinberger (2009b), Tennant (2010), and Steinberger (2011a).

6 Whether harmony is also a *sufficient* condition for logicality is a more delicate question. See Read (2000).

7 See also e.g. Hacking (1979, pp. 237–238) and Dummett (1991, pp. 217–218), and the discussion in Steinberger (2011b). For a recent critical discussion of the requirement of harmony, see Rumfitt (2017).

8 The requirement of purity is compatible with multiple occurrences of the same logical operator within the same rule.

9 See Prawitz (1977, p. 36), Dummett (1991, pp. 296–300) and Tennant (1997, pp. 308–321).

10 I owe this point to Dominic Gregory, to whom I am very much indebted.

11 See e.g. Dummett (1991, p. 299).

12 Sequent calculi axiomatisations of intuitionistic and classical logic are exactly alike, except that classical sequent calculi allow for sequents with multiple premises *and* multiple conclusions. In turn, such sequents can be plausibly interpreted as saying that one may not assert all the antecedents and deny all the succedents, where, again, assertion and denial are both seen as primitive speech acts (Restall 2005).

13 To my knowledge, these rules were first discussed in Murzi (2010, Ch. 7, §4.12) and Murzi and Steinberger (2013, p. 181, fn. 37). For a more recent discussion, see Prawitz (2015, p. 29) and Pereira and Rodriguez (2017, p. 1156).

14 Many thanks to an anonymous referee for suggesting this observation.

15 See Prawitz (1973, p. 243), Read (2000, p. 139), and Negri and von Plato (2001, p. 8).

16 In a recent paper, Lloyd Humberstone and David Makinson argue against justifications of intuitionistic logic based on proof-theoretic properties of basic I- and E-rules (what they call ‘elementary rules’), essentially on the assumption that any acceptable axiomatisation of logic ought to be assertion-based and SET-FMLA (Humberstone and Makinson 2011). As they show, on this assumption, it is not even possible to provide acceptable I-rules for $\lnot$ and $\bot$, let alone harmonious such rules. However, as they also observe, their results do not carry over to a SET-SET$_\emptyset$ framework.

17 It worth noticing that, as an added bonus, the empty set interpretation of $\bot$ also helps to solve Carnap’s so-called Categoricity Problem (see e.g. Carnap 1943), without resorting to multiple-conclusions or rules for denial, and without postulating the existence of a necessarily false sentence, as in Garson (2013).

18 As a referee pointed out to me, a version of $\mathsf{CR}^{hl}$ is discussed in Schroeder-Heister (1981, §18, p. 241 and ff).

19 Given negation introduction and negation elimination, $\vee$-$\hbox {I}_p^*$ and $\vee$-$\hbox {E}_p^*$ are equivalent to $\vee ^*$-I and $\vee ^*$-E, which we have already shown to be interderivable, given $\mathsf{CR}^{hl}$ or some classically equivalent rule, with the standard I- and E-rules for disjunction. There is no need for *ex falso quodlibet*, which is just a special case of $\mathsf{CR}^{hl}$, if we are allowed vacuous discharge of assumptions.

20 Without parentheses,  / -I would allow one to invalidly infer *A*, and then *B*, from a derivation of *B* from *A*.

21 Prawitz’s original proof only covers the cases of $\wedge$ and $\rightarrow$, since, in his system, $\vee$ is defined.

22 See Prawitz (1965, pp. 40–41). Notice that Prawitz’s Lemma on permutative reductions (see Prawitz 1965, pp. 49–51) need not be repeated here, since $\mathbf{Ncp }^{+}$ does not contain general elimination rules such as the standard rule of disjunction elimination.
